# Impact of DOTA Conjugation on Pharmacokinetics and Immunoreactivity of [^177^Lu]Lu-1C1m-Fc, an Anti TEM-1 Fusion Protein Antibody in a TEM-1 Positive Tumor Mouse Model

**DOI:** 10.3390/pharmaceutics13010096

**Published:** 2021-01-13

**Authors:** Judith Anna Delage, Alain Faivre-Chauvet, Jacques Barbet, Julie Katrin Fierle, Niklaus Schaefer, George Coukos, David Viertl, Steven Mark Dunn, Silvano Gnesin, John O. Prior

**Affiliations:** 1 Radiopharmacy Unit, Department of Pharmacy, Lausanne University Hospital and University of Lausanne, CH-1011 Lausanne, Switzerland; 2 CRCINA, INSERM 1232-CNRS ERL 6001, University of Angers, University of Nantes, 44000 Nantes, France; alain.faivre-chauvet@univ-nantes.fr; 3 Groupement d’Intérêt Public Arronax, F-44800 Saint-Herblain, France; jacques.barbet@univ-nantes.fr; 4 LAbCore, Ludwig Institute for Cancer Research, Lausanne University Hospital and University of Lausanne, CH-1066 Epalinges, Switzerland; julie.fierle@unil.ch (J.K.F.); steven.dunn@chuv.ch (S.M.D.); 5 Department of Nuclear Medicine and Molecular Imaging, Lausanne University Hospital and University of Lausanne, CH-1011 Lausanne, Switzerland; niklaus.schaefer@chuv.ch (N.S.); david.viertl@chuv.ch (D.V.); 6 Ludwig Institute for Cancer Research and Department of Oncology, Lausanne University Hospital and University of Lausanne, CH-1011 Lausanne, Switzerland; george.coukos@chuv.ch; 7 Institute of Radiation Physics, Lausanne University Hospital and University of Lausanne, CH-1011 Lausanne, Switzerland; silvano.gnesin@chuv.ch

**Keywords:** TEM-1, fusion protein antibody, DOTA conjugation, ^177^Lu radiolabeling, biodistribution, tumor/liver ratio, theranostic

## Abstract

1C1m-Fc, an anti-tumor endothelial marker 1 (TEM-1) scFv-Fc fusion protein antibody, was previously successfully radiolabeled with ^177^Lu. TEM-1 specific tumor uptake was observed together with a non-saturation dependent liver uptake that could be related to the number of dodecane tetraacetic acid (DOTA) chelator per 1C1m-Fc. The objective of this study was to verify this hypothesis and to find the best DOTA per 1C1m-Fc ratio for theranostic applications. 1C1m-Fc was conjugated with six concentrations of DOTA. High-pressure liquid chromatography, mass spectrometry, immunoreactivity assessment, and biodistribution studies in mice bearing TEM-1 positive tumors were performed. A multi-compartment pharmacokinetic model was used to fit the data and a global pharmacokinetic model was developed to illustrate the effect of liver capture and immunoreactivity loss. Organ absorbed doses in mice were calculated from biodistribution results. A loss of immunoreactivity was observed with the highest DOTA per 1C1m-Fc ratio. Except for the spleen and bone, an increase of DOTA per 1C1m-Fc ratio resulted in an increase of liver uptake and absorbed dose and a decrease of uptake in tumor and other tissues. Pharmacokinetic models correlated these results. The number of DOTA per antibody played a determining role in tumor targeting. One DOTA per 1C1m-Fc gave the best pharmacokinetic behavior for a future translation of [^177^Lu]Lu-1C1m-Fc in patients.

## 1. Introduction

Radiolabeled monoclonal antibodies (mAbs) have been actively investigated for theranostic applications [1]. The radiolabeling of a mAb with a metallic radionuclide, generally involves the use of suitable bifunctional chelating agents (BFCAs) with high metal-chelate stability constants. BFCAs are designed to stably coordinate the radionuclide and to allow a covalent attachment to protein functional groups [2,3]. Most protocols used to conjugate antibodies with BFCAs are not site-specific and result in a variable number of BFCAs per antibody, depending on experimental conditions and antibodies themselves. With non-site-specific processes, the average number of BFCA attached per antibody depends upon the molar ratios of antibody and BFCA used for the conjugation as well as on the reaction conditions employed for the conjugation [4].

Dodecane tetraacetic acid (DOTA) derivatives, which are hydrophilic macrocyclic ligands, have been used as the most popular BFCAs for the development of radio-lanthanide-labeled mAbs [5].

An increasing chelator-to-antibody ratio often allows to improve the specific activity of the radiolabeled compound. Nevertheless, the hydrophilicity/lipophilicity, the charges of the conjugate, and consequently the pharmacokinetics of the antibody can be modified by the conjugation of hydrophilic DOTA chelator [4].

Authors showed that an increasing number of DOTA per antibody resulted in a decrease of the non-specific liver uptake [6,7]. The provided explanation was the reduction of the isoelectric point (pI) correlated to the increase of the negative charge given by the DOTA chelator resulting in important repulsion between the lipid bilayer and the conjugate. However, the impact of the increasing number of negative charges on the biodistribution was unclear. On the opposite, some groups observed a rapid blood clearance, a decrease of the tumor uptake and an increase of the hepatic uptake with high number of chelators conjugated to an antibody [8,9]. The conjugation with a high number of DOTA can alter the immunological properties of the antibody due to the possibility of DOTA to bind the variable domains of the antibody, involved in antigen targeting [4]. Moreover, a high number of chelators per antibody could change the tumor targeting pharmacokinetic due to the uptake of the conjugate by the reticuloendothelial system in liver and spleen [1].

The biodistribution of radiolabeled conjugated antibody is determined by the chelator to antibody ratio but also by many different parameters of the radionuclide such as the size, the chelation geometry and the coordination number. It would be necessary to optimize the conjugate regarding these criteria [6,8].

In this study, 1C1m-Fc, a scFv-Fc fusion antibody constructs which bind to murine and human tumor endothelial marker 1 (TEM-1) was conjugated to p-SCN-Bn-DOTA chelator. After conjugation 1C1m-Fc was radiolabeled with ^177^Lu. This radionuclide, which is a γ and β^−^ emitter allowing theranostic approach.

TEM-1, also named endosialin/CD248, is a 80.9 kDa type I cell surface transmembrane protein of the C-lectin receptor family [10,11,12] implicated in development, vascular cell adhesion and migration, neoangiogenesis, and tumor progression [13,14]. TEM-1 over expression correlates with a poor patient prognosis and a tumor aggressiveness [15,16].

Its high expression on the tumor vasculature of several solid human cancers, with limited expression in normal adult tissue, makes TEM-an ideal target for theranostic applications [17,18].

Our previous study showed that [^177^Lu]Lu-1C1m-Fc could prove as a potentially useful and safe tool for theranostic applications [19]. In these experiments, while the TEM-1 positive uptake was specific, we also observed an important liver uptake that was not saturation-dependent. Our hypothesis for this phenomenon was the influence of the number of DOTA on the biodistribution.

The conjugation of antibodies and antibody fragments with chelator plays a significant role in determining the success of tumor targeting employing radiolabeled antibodies [4,8]. Therefore, the goal of this study was to evaluate the effect of coupling an increasing number of DOTA per 1C1m-Fc on the pharmacokinetic behavior, immunoreactivity, and dosimetry of the radiolabeled antibody complex to develop an optimal radiolabeled 1C1m-Fc suitable for theranostic application.

## 2. Materials and Methods

### 2.1. Fusion Protein Antibody

Complete description of the single-chain variable fragment (scFv) 1C1m-Fc (Molecular Weight = 106196.8 Da) was done in Delage et al [19] and Fierle et al [20]. Briefly, this fusion protein antibody recognizes efficiently human and murine TEM-1 antigen over expressed in tumor cells and in SK-N-AS cell line that was chosen to develop the animal model.

### 2.2. Cell Lines

The human neuroblastoma SK-N-AS (TEM-1 positive) cell lines was purchased from American Type Culture Collection (ATCC, Manassas, VA, USA).

SK-N-AS cells were cultured in DMEM (Thermo Fisher Scientific, Waltham, MA, USA) supplemented with 0.1 mM Non-Essential Amino Acids (Thermo Fisher Scientific, Waltham, MA, USA), 10% fetal bovine serum (FBS, Thermo Fisher Scientific, Waltham, MA, USA) and 1% penicillin/streptomycin (Thermo Fisher Scientific, Waltham, MA, USA). Cells were incubated at 37 °C in a humidified atmosphere at 5% CO_2_.

### 2.3. Conjugation

Antibody concentration was measured at 280 nm using a spectrophotometer (NanoDrop Lite, Thermo Fisher Scientific, Waltham, MA, USA). To obtain conjugates with increasing ligand-to-antibody ratios, 6 concentrations of p-SCN-Bn-DOTA (Macrocyclics, Plano, TX, USA; MW: 551.6) from 5 to 50 equivalents were used.

Prior to the coupling procedure, the 1C1m-Fc was conditioned in carbonate buffer 0.2 M pH 9.0 by ultrafiltration on a 50 kDa ultrafiltration membrane (Amicon Ultra, 0.5 mL, 50 kDa, Merck, Darmstadt, Germany). To 1 mg (9.4 nmol; 200 µL) of 1C1m-Fc was added a calculated quantity of a 25.9 mg/mL (47 µmol/mL) p-SCN-Bn-DOTA solution in an extemporaneously made mixture of 10% DMSO (*v*/*v*) in the same carbonate buffer. The BFCA-to-1C1m-Fc ratios used were 5, 10, 20, 30, 40, and 50.

Antibody coupling solutions were incubated for 1 h at 37 °C and the conjugated antibodies were washed by four ultrafiltrations using PBS pH 7.4 before performing high-pressure liquid chromatography (HPLC) to assess integrity of the conjugates. Conjugated fusion protein antibodies were subsequently stored between 2 and 8 °C.

### 2.4. Mass Spectrometry Analysis

Mass spectrometry (MS) analysis was performed using a Q Exactive HF Orbitrap (Thermo Fisher Scientific, Waltham, MA, USA) and separation was done using a MAbPAC SEC-1 column, (Thermo Fisher Scientific, Waltham, MA, USA) with a mobile phase of ammonium acetate 50 mM pH 7.0 at 0.3 mL/min as previously described [19]. After deconvolution of the mass spectrometry spectra, the drug-to-antibody ratio (DAR) is calculated using the formula:

Σ(n*Int)/Σ (Int)

where *n* = number of attached molecules for this peak and Int = intensity of the peak.

### 2.5. Radiolabeling

The radiolabeling was optimized in acetate buffer 0.4 M pH 5.6 with respectively 500 pmol of DOTA-conjugated 1C1m-Fc and 20 MBq of ^177^Lu without carrier in aqueous 0.04 M HCl solution (EndoleucineBeta 40 GBq/mL, ITM, Garching bei München, Germany). After 1 h incubation time at 37 °C, the radiochemical purity was determined by instant thin layer chromatography (iTLC) in citrate buffer 0.1 M pH 5.0.

The release criterion was radiochemical purity over 95%.

If necessary, the excess of ^177^Lu was removed with one to three ultrafiltrations on 50 kDa membrane (Amicon Ultra, 0.5 mL, 50 kDa, Merck, Darmstadt, Germany) in acetate buffer 0.4 M pH 5.6.

### 2.6. Purity and Stability

Chemical purity of 1C1m-Fc was tested using HPLC and gel electrophoresis as described in Delage et al. [19]. Stability of the fusion protein was evaluated at 3, 6, and 12 months after his production by HPLC only. Radiochemical purity after antibody radiolabelling was assessed by TLC on iTLC-SG at 24 and 48 h.

#### 2.6.1. HPLC

As described in Delage et al. [19], HPLC analyses were done using an Ultimate 3000 SD System (Thermo Fisher Scientific, Waltham, MA, USA) and a GabiStar radiodetector (Elysia-Raytest GmBH, Straubenhard, Germany). A size exclusion chromatography was performed using phosphate buffer pH 6.8 as solvent and a 200 kDa size exclusion column (XBridge protein BEH, Waters, Baden-Dättwil, Switzerland). Each chromatography profile was analyzed at 280 nm.

#### 2.6.2. iTLC

TLC on iTLC-SG (Agilent Technologies, Folsom, CA, USA) was performed in citrate buffer 0.1 M pH 5.0. Using these conditions, unbound ^177^Lu is complexed by the solvent and migrates at retention factor (*Rf*) = 1 while charged [^177^Lu]Lu-1C1m-Fc remains at *Rf* = 0.

### 2.7. In Vitro Characterization of Immunoreactivity

Immunoreactive fraction assessment was done as in Delage et al. [19]. Briefly, each coupled 1C1m-Fc-DOTA and native 1C1m-Fc were evaluated by Lindmo assay [21]. An increasing number of SK-N-AS cells (0.25–8 × 10^6^) were incubated with a fixed concentration of radiolabeled 1C1m-Fc (0.07 µg/mL; 0.659 pmol/mL). A fusion protein antibody excess of 100-fold concentration was used to evaluate the non-specific binding. The immunoreactive fraction was calculated by extrapolation to an infinite cells number by fitting the curve with a non-linear regression method (Graphpad Prism 8.0, 2018 GraphPad Software, San Diego, CA, USA).

### 2.8. In Vivo Characterization

#### 2.8.1. Murine Xenograft Model

All animal experiments were performed in accordance with the Swiss legislation for the care and use of laboratory animals under the license VD-2993 (09/2018) delivered after approbation by the Veterinarian Office of the canton of Vaud and the ethics committee.

Female Balb/C nude mice (Charles River Laboratories, Wilmington, MA, USA) between 8 and 10 weeks were subcutaneously grafted with 3.00 × 10^6^ SK-N-AS cells as described in Delage et al. [19]. Mice were assigned to the experimental groups when the tumor reached 5–10 mm diameter size.

#### 2.8.2. Biodistribution Studies

To define the impact of the conjugation on the biodistribution, a mixture of 2.5 µg (23.5 pmol) of [^177^Lu]Lu-1C1m-Fc conjugated with respectively 1, 2.5, 3, 6, 8, and 11 DOTA per 1C1m-Fc and 47.5 µg (447.3 pmol) of native unlabeled 1C1m-Fc was injected into the lateral tail vein of the mice (*n* = 3) without anesthesia. The volume for all the injections was 100 µL and sodium chloride 0.9% (B.Braun, Sempach, Switzerland) was used to perform the dilution. The injected solution was not filtered.

The average weight of animals was 18.4 ± 1.8 g. The dose of 50 µg (470 pmol) of antibody has been selected from our previous study [19].

Mice were sacrificed by CO2 inhalation 24 h after radiolabeled antibody injection. Blood was collected by exsanguination. Organs and tumors were weighted after drying and them and counted with a gamma counter (AMG Automatic Gamma Counter, Hidex, Turku, Finland).

For the [^177^Lu]Lu-1C1m-Fc conjugated with 1 and 3 DOTA, complementary time points have been added for the biodistribution, and animals (*n* = 3) were euthanized 4, 24, 48, 72 h, and 6 days after injection.

Results were expressed as a percentage of injected activity (IA) per gram of tissue (%IA/g).

#### 2.8.3. Pharmacokinetic Modeling

Data were expressed as percent injected activity per gram of tissues. A multi-compartment pharmacokinetic model was used in which the injected antibody was distributed from a central compartment, representing the blood, into peripheric compartments corresponding to all investigated organs plus an additional compartment representing all uncounted tissues. Tissue contents were calculated as the content of the tissue compartment plus a fraction of blood activity. This is equivalent to consider fast and a slow distribution compartments as in similar models [22] given that the fast kinetics cannot be accounted for from data collected over 6 days. The biodistribution kinetics for all studied tissues were modelled for the 1 and 3 DOTA per 1C1m-Fc using a software package developed in Arronax Laboratory available upon request (www.arronax-nantes.fr). This software package, similar to and validated by comparison with WinSAAM [23], allows pharmacokinetic modelling directly from a Microsoft Excel worksheet. Differential equations were solved numerically using the Chu–Berman algorithm [24]. Variable parameters were estimated using the non-linear weighted least squares Levenberg–Marquardt algorithm.

**Figure A1:**
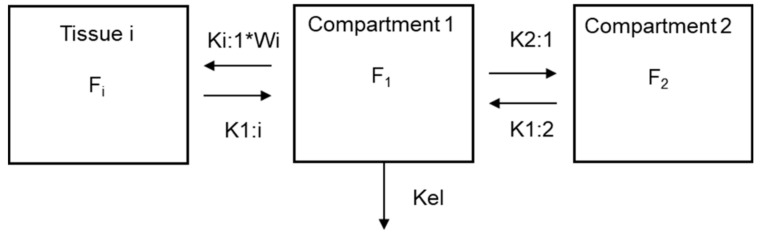
Multiple compartment mathematical model.

**Figure A2:**
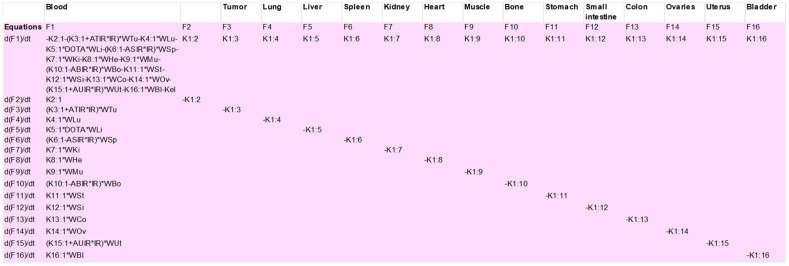
Excel data sheet example of a multi-tissue biodistribution (16 compartments) modeling.

**Figure A3:**
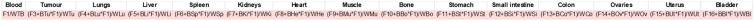
Example of formulas used for modeling each measured tissue.

It was then assumed that the rate of liver uptake was proportional to the number of DOTA per antibody and that the rates of uptake into tumor and uterus (a normal tissue expressing low amounts of antigen) increased linearly with the immunoreactivity. Conversely, the rates of spleen and bone uptake were assumed to decrease linearly with the immunoreactivity. Then all available biodistribution data, at all time-points for 1 and 3 DOTA per 1C1m-Fc, and at 24 h after injection for the other conjugates were fitted simultaneously using a single set of kinetic parameters. The model is described in Appendix A (Figure A1, Figure A2 and Figure A3 and Table A1.).

#### 2.8.4. Murine Dosimetry

Estimated absorbed doses to organs were based on the biodistribution results of mice bearing TEM-1 positive tumor injected with [^177^Lu]Lu-1C1m-Fc conjugated with 1 DOTA. Considered source organs were liver, kidneys, lungs, spleen, heart (cardiac muscle), blood pool, stomach, small intestine, colon, ovaries, uterus, urinary bladder, salivary glands, and the total body. The reminder was obtained by subtraction of the signal measured in source organs from the total body. For each mouse at each time point, the activity in each source organ and the remainder was normalized by the total injected activity to obtain the normalized injected activity (nA). For each source organ at each time point, an average nA value was obtained ±SD.

For all source organs with the exception of stomach, uterus, salivary glands and the urinary bladder, the normalized time activity curves (nTACs) were fitted with bi-exponential functions using the kinetic module of OLINDA/EXM 2.1 (HERMES Medical Solution AB, Stockholm, Sweden). Time-integrated activity coefficients (TIACs) were derived by analytical time-integration of fitted source organ nTACs obtained with the average nA, nA + SD and the nA − SD values, respectively.

The nTACs for stomach, uterus, salivary glands, urinary bladder and the tumor were not conveniently fitted by monotonically decreasing bi-exponential functions. For these tissues, the TIAC was obtained by trapezoidal integration using Matlab software (Release 2019b, The MathWorks, Inc., Natick, MA, USA), between *t* = 0 and *t* = 6 days, whereas a mono-exponential analytical integration to infinity was calculated after the last measure (*t* > 6 days) considering the ^177^Lu physical decay constant.

Finally, source organ TIACs were entered into the OLINDA/EXM^®^ 2.1 software kinetic module for organ absorbed dose estimates considering the 25 g murine model where the phantom source organ masses were adjusted to the average organ masses obtained from the mice population considered for the dosimetry experiment. In this process, the TIAC of the ovaries, uterus and the salivary glands was part of the remainder of the body.

A specific absorbed dose estimation was obtained for ovaries, uterus and the salivary glands. These organs, in fact, exhibit an important specific tracer uptake, but were not among the source/target organs available in the murine model of OLINDA/EXM 2.1 software. For these organs, the absorbed dose estimation was obtained using the sphere model of OLINDA/EXM 2.1 where the average organ TIAC and the average organ mass were applied.

Estimated absorbed doses to tumor and selected organs based on the biodistribution results on TEM-1 positive tumor bearing mice injected with [^177^Lu]Lu-1C1m-Fc conjugated respectively with 1 and 3 DOTA were compared. The dosimetry with the 3 DOTA conjugation was obtained from our previous study [19]. The selected organs were the liver, the lungs, the kidney, the spleen and the uterus.

### 2.9. Statistics

The data are expressed as mean ± SD (standard deviation) or SEM (standard error to the mean). Significant differences between immunoreactive fractions were analyzed by ordinary one-way Anova using the Turkey’s multiple comparisons method. Data from biodistribution studies were analyzed by an unpaired, 2-tailed Student t test with a correction for multiple comparison using the Holm–Sidak method (α = 0.05). Correlation between the tumor/liver ratio and the ratio of DOTA per 1Cm-Fc were analyzed with a Spearman test. Curve-fitting and statistical analyses were conducted using Prism 8.0 (GraphPad Software, San Diego, CA, USA). Pharmacokinetics analyses were performed with Kinetics software.

## 3. Results

### 3.1. Conjugation and Radiolabeling

1C1m-Fc was conjugated with six concentrations of DOTA between 5 and 50 equivalents. The number of DOTA was estimated for each concentration (Table 1, Figure S1) and was between 1 and 11 DOTA.

1C1m-Fc and its conjugates were analyzed by HPLC. The purity of conjugated antibodies is reported in Table 1. The HPLC profiles, the stability of the native and conjugated fusion protein antibody, and the stability in serum of [^177^Lu]Lu-1C1m-Fc were given in our prior publication [19].

The release criteria for the radiochemical purity (RCP) evaluated by TLC was more than 95%. To reach this criterion with antibodies modified with 1 and 2.5 DOTA, ultrafiltration on amicon membrane (Amicon Ultra, 0.5 mL, 50 kDa, Merck, Darmstadt, Germany) was used. HPLC was not used in this study to evaluate the RCP as this test was done in our previous study [19] and the results were similar to that obtained using TLC.

### 3.2. Immunoreactive Fraction

The immunoreactivity following the radiolabeling was assessed by Lindmo assay (Table 2; Figure S2).

For validation tests comparative immunoreactivity assessment with incubation at 37 °C and 4 °C were carried out and the results obtained showed no difference at 3 h. Furthermore, internalization results of 1C1m-Fc radiolabeled with ^125^I have been published and showed that the rate of internalization was quite slow suggesting that the antibody does not trigger the rapid migration of TEM-1 from the cell surface [25]. The immunoreactivity, that was 85.1 ± 1.3, 86.2 ± 2.7, 87.5 ± 1.0, and 78 ± 1.4% for 1, 3, 6, and 8.5 DOTA, respectively suggesting that it was not affected by the conjugation up to 8.5 DOTA (Turkey’s multiple comparisons test, *p* > 0.068, *n* = 17).

On the other hand, a significative loss of immunoreactivity to 24 ± 1.7% was obtained with the highest number of BFCA (11 DOTA per fusion protein antibody) compared to the others ratios (Turkey’s multiple comparisons test, *p* < 0.0001, *n* = 17).

### 3.3. In Vivo Characterization

#### 3.3.1. Biodistribution Study at 24 h

The biodistribution of [^177^Lu]Lu-1C1m-Fc conjugated with 1, 2.5, 3, 6, 8, and 11 DOTA units respectively was performed 24 h after injection.

**Figure 1:**
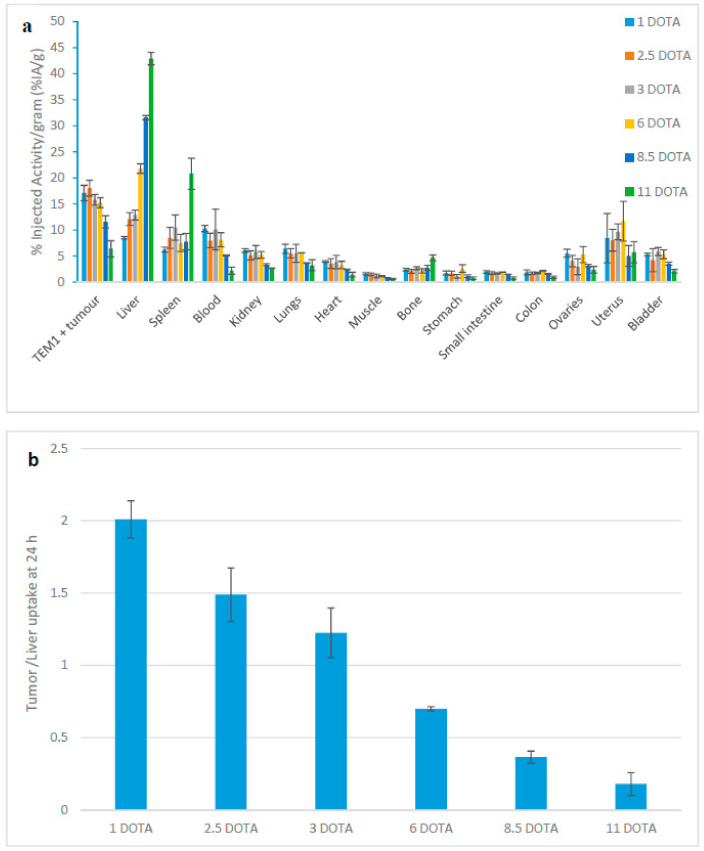
(**a**) Biodistribution at 24 h of [^177^Lu]Lu-1C1m-Fc conjugated with 1 to 11 DOTA in Balb/c nu mice bearing TEM-1 positive tumor. Data are shown as mean ± SD. (**b**) Ratio between the tumor and the liver uptake at 24 h with respect to the number of DOTA per [^177^Lu]Lu-1C1m-Fc in Balb/c mice bearing TEM-1 positive tumor. Spearman test gives a rho = −0.99, *p* < 0.0001.

A decrease of tumor uptake was observed with the 1C1m-Fc conjugated with more than 3 DOTA (18.8 ± 1.5% IA/g up to 3 DOTA to 5.3 ± 1.6% IA/g for 11 DOTA). In parallel, an accelerated blood clearance was observed with the increasing number of chelator and the radiotracer circulating in the blood at 24 h varied from 10.2 ± 0.6% for 1 DOTA per antibody to 2.2 ± 0.7% for 11 DOTA per antibody (Figure 1a).

An inverse correlation of the tumor/liver ratio was observed with the increasing number of DOTA per antibody, from 2 with 1 DOTA per antibody to 0.15 with 11 DOTA per antibody (Spearman test, rho = −0.99, *p* < 0.0001) (Figure 1b).

#### 3.3.2. Complementary Analyses for 1C1m-(DOTA)_1_ and 1C1m-(DOTA)_3_

For the [^177^Lu]Lu-1C1m-Fc conjugated with 1 and 3 DOTA, complementary time points have been added for the biodistribution and animals were euthanized at 4, 24, 48, 72 h, and six days after injection.

**Figure 2:**
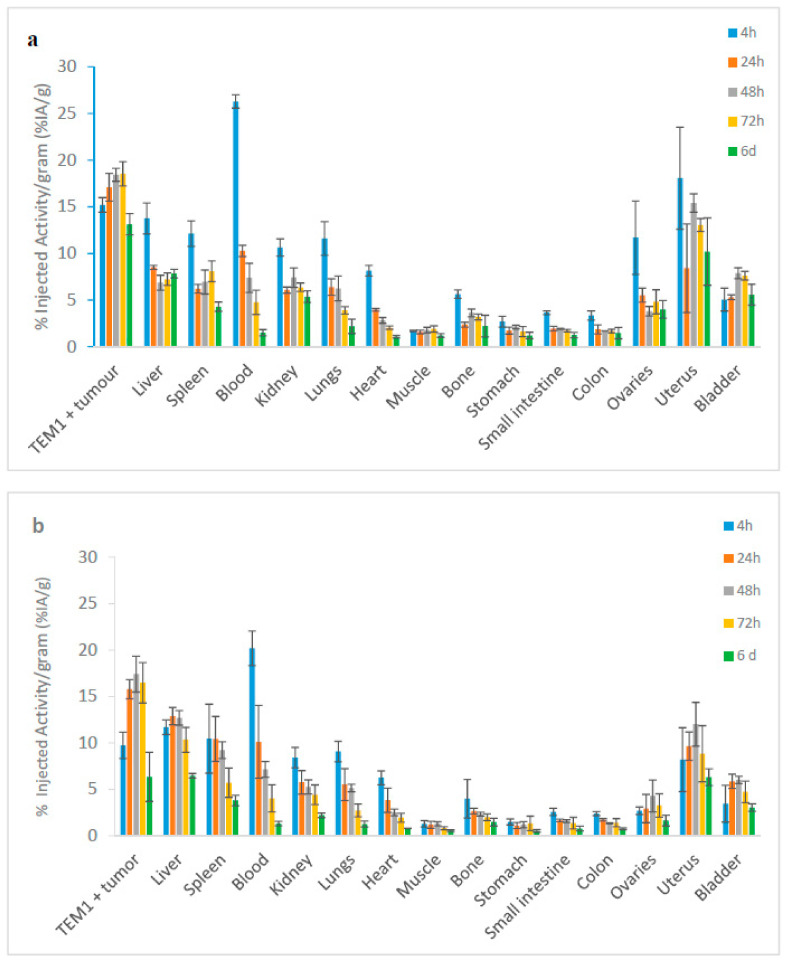
Biodistribution of [^177^Lu]Lu-1C1m-Fc in Balb/c nu mice bearing TEM-1 positive tumor, (**a**) conjugated with 1 DOTA; (**b**) conjugated with 3 DOTA. Data are shown as mean ± SD, (*n* = 3).

The uptake in TEM-1 positive tumors was unchanged between the two groups. However, in the case of [^177^Lu]Lu-1C1m-Fc conjugated with 1 DOTA, the non-specific uptake in the liver was lower than that observed with 3 DOTA conjugated at 24 and 48 h, where *p* = 0.02 and 0.01 (unpaired t-test, *n* = 3) respectively (Figure 2a,b).

#### 3.3.3. Pharmacokinetic Modeling

Kinetics with 1C1m-Fc conjugated respectively with 1 and 3 DOTA were satisfactorily fitted by the model (Figure S3a,b).

Tissues showing highest uptake were the tumor and the uterus and, for 1C1m-Fc conjugated with 3 DOTA, the liver. For the liver, the estimated uptake rate constants of the 1C1m-Fc conjugated with 3 DOTA was 3.5 times that of the 1 DOTA, in line with the higher uptake. The wash-out rate was relatively fast for the 3 DOTA, but, because of a single high value at six days, was fitted to 0 for the 1 DOTA, preventing further comparison. The differences in estimated rate constants and tissue blood contents for the other tissues were hard to interpret because of relatively high SD on measurements, particularly for uterus and bone.

As expected, the simultaneous fit (Figure S4) represented less closely the biodistribution data, but the general shape and trends were conserved.

**Figure 3:**
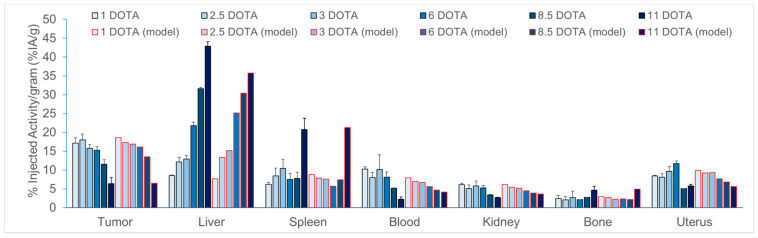
Comparison between the results obtained by biodistribution (in grey) and pharmacokinetic modeling (in red) at 24 h for [^177^Lu]Lu-1C1m-Fc conjugated with 1 to 11 DOTA in Balb/c nu mice bearing TEM-1 positive tumor.

More interestingly, the trends in 24 h biodistributions for the six different concentrations of DOTA were well replicated (Figure 3).

The increased liver uptake at higher numbers of DOTA effectively decreases the amount of circulating [^177^Lu]Lu-1C1m-Fc and consequently the amount of [^177^Lu]Lu-1C1m-Fc in most of other organs. The loss of immunoreactivity explains the decrease of the TEM-1 specific uptake in the tumor and the uterus, especially at the two highest DOTA per antibody ratios. Finally, the increase of the spleen and bone uptake at the highest concentrations of DOTA was accounted by a higher uptake of non-immunoreactive [^177^Lu]Lu-1C1m-Fc. This condition was simulated by a linear decrease of the spleen uptake rate with immunoreactivity. 

#### 3.3.4. Murine Dosimetry

Extrapolated organ absorbed doses for mice derived from the injection of [^177^Lu]Lu-1C1m-Fc conjugated with 1 DOTA are given in Table 3. The organs receiving the highest absorbed dose was the uterus (1.83 ± 0.14 Gy/MBq), followed by the liver (1.79 ± 0.13 Gy/MBq), the stomach wall (1.66 ± 0.08 Gy/MBq) and the kidneys (1.32 ± 0.05 Gy/MBq). The total body dose was 0.55 ± 0.04 Gy/MBq and the tumor dose was 2.53 ± 0.25 Gy/MBq. The tumor-to-liver absorbed dose ratio was 1.41.

The absorbed doses for tumor, liver, kidneys, lungs, uterus and bladder were compared between [^177^Lu]Lu-1C1m-Fc conjugated respectively with 1 DOTA and 3 DOTA (Table 4).

The tumor/liver absorbed dose ratio increased from 0.8 for the [^177^Lu]Lu-1C1m-Fc conjugated to 3 DOTA to 1.4 for the [^177^Lu]Lu-1C1m-Fc conjugated to 1 DOTA. The non-specific uptake in the kidneys, the lungs and the specific uterus uptake was higher with the fusion protein conjugated with 1 DOTA.

## 4. Discussion

Because of its expression across many tumors, its low expression in normal tissues and accessibility from the vascular circulation, TEM-1 is emerging as an interesting biomarker for theranostics [26]. Several IgG antibodies targeting the lectin-like domain of TEM-1 have already been developed for oncological application [13,26].

Given the very short half-life and the relative in vivo instability of monovalent scFv antibody fragments, a bivalent Fc-fusion protein based on a novel single chain antibody, 1C1m-Fc, has been synthesized. The fusion of scFvs to the IgG Fc constant domains adds significant size, avidity and stability to the targeting moiety and would be expected to lead to improved blood pharmacokinetics.

Our previous study showed the relevance of this novel fusion protein antibody radiolabeled with ^177^Lu for a theranostic approach [19]. The aim of the present work was to study the effect of the DOTA conjugation on the immunoreactivity, the pharmacokinetics and the dosimetry of [^177^Lu]Lu-1C1m-Fc.

Six different conjugates were obtained by incubating 1C1m-Fc with several molar ratio of DOTA respectively: 5, 10, 20, 30, 40, and 50 equivalents of DOTA. All the conjugates were analyzed by mass spectrometry and the number of DOTA moieties attached per 1C1m-Fc were respectively 1, 2.5, 3, 6, 8.5, and 11. Even if the HPLC profile of these conjugates was similar, they are expected to have different pharmacokinetics behavior.

Radiolabeling was performed with ^177^Lu to obtain formulation with a RCP of more than 95%. The immunoreactivity following the radiolabeling was assessed by Lindmo assay. The immunoreactivity was not affected by the conjugation up to 8.5 DOTA. Nevertheless, a significative loss of the immunoreactivity was observed with 11 DOTA (IR = 24%). Several studies indicated that immunoreactivities of radiolabeled antibody were getting compromised with the increase in the number of BFCA attached per antibody moieties. Indeed, conjugation of the variable chain can weaken or abrogate antigen binding which in turn decreases the efficacy of the targeting of the immunoconjugate [27,28]. Wangler et al. demonstrated that the size of the conjugated dendritic structure does not significantly influence the immunoreactivity of the antibodies over a wide molecular weight range, whereas the number of derivatization sites is the major factor that determines the binding affinity of the conjugates [29]. Grunberg et al. and Fischer et al. [28,30] showed that an enzymatic conjugation leads to immunoconjugates with a uniform and well-defined substitution only on the heavy chain. With this technique increasing numbers of DOTA moieties was accompanied by an increasing specific activity of the immunoconjugates when labeled with ^177^Lu. The advantage of the high specific activity was not counteracted by the simultaneous decrease of immunoreactivity. A site-specific enzymatic conjugation to the constant region could be better by less altered radio-immunoreactivity [31].

A biodistribution study of [^177^Lu]Lu-1C1m-Fc conjugated with all the DOTA conjugates was performed. A significant decrease of the tumor uptake was observed 24 h after injection with the 1C1m-Fc conjugated with more than 3 DOTA. This time point has been chosen as we have seen in our previous study that it was the most informative one [19]. This behavior could be attributed to the increased hydrophilicity of [^177^Lu]Lu-1C1m-Fc with the number of DOTA attached to the molecule. Indeed, highest number of hydrophilic DOTA or chelator has been described to exhibit a rapid blood clearance resulted in an increasing uptake in the liver [4,8,32,33], but the mechanism was unclear. Knogler et al. proposed that it can be due to the conformational change of the backbone structure of the antibody induced by over-coupling, resulting in a rapid sequestration by the reticuloendothelial system in the liver but invalidated this hypothesis as no difference was found in CD spectra between substituted and unsubstituted antibody [1,8].

On the other hand, it has been suggested that the negative charge conferred to the antibody by DOTA conjugation results in a reduced isoelectric point (pI), causing a net repulsion between the molecule and the phospholipid bilayer, reducing the hepatobiliary excretion or the hepatic uptake [6,7]. Several publications indicate that a decrease of the liver uptake could be observed with negatively charged peptides or antibodies derivates compared to neutral or positively charged conjugated variants [9,34,35,36]. General approach described to improve imaging contrast in the liver include increasing the hydrophilicity via a hydrophilic chelator or linker, modifying the positioning and composition of potential purification tags or increasing negative charge [34,35,36]. These observations differ to the results of our experiment. However, it is relevant to note that, in these publications, different types of chelates grafted on same vector were compared but with the same chelate-to-vector ratio. The impact of the different chelators on biodistribution and imaging contrast was assessed. On the contrary, we have studied the effect of a same chelate, DOTA, with various antibody-to-ligands ratios. This difference in the methodology could explain the different results.

Complementary analysis has been added for 1 and 3 DOTA to ensure the consistency of the model. The biodistribution of the radiolabeled antibody was well described using a multi-compartment model that showed a clear increase in the liver uptake rate between 1 and 3 DOTA per antibody. To further rationalize this effect, all available data were fitted simultaneously using the same compartment model, assuming linear relationships between the liver uptake rate constant and the number of DOTA and between the tumor and uterus uptake rate constants and the immunoreactivity. Finally, uptake in the spleen and the bone was assumed to increase with the loss of immunoreactivity. Linear relationships were selected as first order approximations. This model was consistent with the observed data: increased liver uptake at higher DOTA-substitution ratios depletes the circulating antibody and the amount of antibody found in all tissues. In addition, the loss of immunoreactivity further decreases the specific absorption into the tumor and uterus. Assuming a faster uptake of non-immunoreactive antibody in spleen and bone accounts for the high uptake seen with the 11-DOTA antibody. While a model cannot be considered a proof, this one shows that simple hypotheses may explain the observations made in biodistribution experiments.

We decided to evaluate the extrapolated organ absorbed doses for mice derived from the injection of [^177^Lu]Lu-1C1m-Fc conjugated with the two lowest concentrations of DOTA, namely 1 and 3 DOTA per fusion protein antibody as they gave the best specific/non-specific uptake ratio in the biodistribution study. Organ receiving the highest doses were liver and uterus. Two other anti TEM-1 antibodies, 78Fc labeled with ^111^In and Morab-004 labeled with ^124^I showed similar results in these organs [26,37].

The tumor/liver absorbed dose ratio increased from 0.8 for the [^177^Lu]Lu-1C1m-Fc conjugated to 3 DOTA to 1.4 for the [^177^Lu]Lu-1C1m-Fc conjugated to 1 DOTA. The absorbed dose ratio tumor/liver was multiplied by 1.75 with the [^177^Lu]Lu-1C1m-Fc conjugated to 1 DOTA compared to 3 DOTA.

Even if the theorical and experimental specific activity for 3 DOTA is higher than for 1 DOTA (experimentally 400 MBq/mg vs. 200 MBq/mg; data not shown), this difference has not been taken into account in this study considering it small influence in therapeutic applications. Indeed, regarding the professional practices in radioimmunotherapy the amount of antibody usually injected in human is comprised between 1 and 1.5 mg/kg. If we consider the lowest specific activity obtained with 1 DOTA, the quantity of antibody injected will be sufficient to reach more than 8 GBq for all patients of more than 45 kg weight. Therefore, 1C1m-Fc appeared as a very promising compound for a theranostic approach.

## 5. Conclusions

Antibody labeling with metal radionuclides requires the use of a bifunctional chelator to attach radioactive metal to the protein, ideally without affecting the pharmacokinetics of the antibody [34]. In our experiments, we have demonstrated that the number of chelators per fusion protein antibody plays a significant role in determining successful tumor targeting. There is thus an opportunity to further improve the biodistribution and imaging contrast. Both absolute tumor uptake and target-to-non target ratios are important for the selection of the best imaging agent [35]. In this study, [^177^Lu]Lu-1C1m-Fc conjugated with 1 DOTA was to be the best ratio to maintain a balance between the specific activity, immunoreactivity, and pharmacokinetic behavior and appears as an interesting candidate for further theranostic development.

## 6. Patents

J.K.F, S.M.D. and G.C. hold patents in the domain of antibodies and in particular on the 1C1m antibody used in this study.

## Figures and Tables

**Table 1:** Estimated number of DOTA per 1C1m-Fc based on mass spectrometry and purity analyses of the conjugates from 5 to 50 equivalents (eq) of DOTA. The estimated DAR is calculated using the formula: Σ(n*Int)/Σ (Int), where *n* = number of attached molecules for this peak, Int = intensity of the peak.

Compound	Mass Weight (Da)	Estimated Number of DOTA per 1C1m-Fc	% Purity (HPLC)
Unmodified 1C1m-Fc	108,394	NA (not applicable)	97.4%
DOTA (- HCl-H_2_O)	551	NA	NA
1C1m-Fc 5 eq DOTA	108,395–108,985	1	95.6%
1C1m-Fc 10 eq DOTA	108,986–110,758	2.5	96.2%
1C1m-Fc 20 eq DOTA	109,496–111,746	3	95.7%
1C1m-Fc 30 eq DOTA	110,755–113,117	6	96.9%
1C1m-Fc 40 eq DOTA	111,746–114,664	8.5	96.2%
1C1m-Fc 50 eq DOTA	113,711–116,068	11	96.8%

**Table 2:** Radioimmunoreactive fraction results for [^177^Lu]Lu-1C1m-Fc conjugated with 1 to 11 DOTA.

Number of DOTA per 1C1m-Fc	Immunoreactivity (%) ± SEM
1	85.1 ± 1.3
3	86.2 ± 2.7
6	87.5 ± 1.0
8.5	78 ± 1.4
11	24 ± 1.7

**Table 3:** Considered organ masses, estimated source organ TIAC and organ absorbed doses [^177^Lu]Lu-1C1m-Fc. Organ masses of the 25g mouse model of Olinda/EXM 2.1 were used for: brain, thyroid, testes, skeleton, pancreas and the heart content, for all other organs we used the experimental mean masses.

Organ	Mean Organ Mass (g)	TIAC (MBq·h/MBq)		Abs. Dose (mGy/MBq)	
		Mean	SD	Mean	SD
Brain ^t^	0.50	-	-	4.08 × 10^2^	2.70 × 10^1^
Large intestine ^s,t^	0.78	1.98	0.33	7.03 × 10^2^	8.10 × 10^1^
Small intestine ^s,t^	1.20	3.33	0.25	5.77 × 10^2^	4.00 × 10^1^
Stomach ^s,t^	0.26	0.77	0.03	1.66 × 10^3^	8.00 × 10^1^
Heart ^t^	0.11	0.48	0.06	1.10 × 10^3^	1.50 × 10^2^
Heart content ^s^	0.2	1.84	0.35		
Kidneys ^s,t^	0.31	3.37	0.13	1.32 × 10^3^	5.00 × 10^1^
Liver ^s,t^	1.13	21.49	1.72	1.79 × 10^3^	1.30 × 10^2^
Lungs ^s,t^	0.15	1.03	0.31	9.83 × 10^2^	2.07 × 10^2^
Pancreas ^t^	0.30			4.41 × 10^2^	2.80 × 10^1^
Skeleton ^t^	2.20			4.18 × 10^2^	2.80 × 10^1^
Spleen ^s,t^	0.10	1.06	0.03	1.18 × 10^3^	1.00 × 10^2^
Ovaries ^s,^*	0.04	0.41	0.08	7.42 × 10^2^	9.90 × 10^1^
Uterus ^s,^*	0.11	2.54	0.24	1.83 × 10^3^	1.40 × 10^2^
Testes ^t^	0.16			4.09 × 10^2^	2.60 × 10^1^
Thyroid ^t^	0.01			4.09 × 10^2^	2.70 × 10^1^
Salivary glands ^s,^*	0.11	0.75	0.02	5.41 × 10^2^	1.70 × 10^1^
Urinary Bladder ^s,t^	0.02	0.17	0.01	5.34 × 10^2^	3.70 × 10^1^
Total Body ^s,t^	18.44	111.08	6.54	5.49 × 10^2^	3.80 × 10^1^
Tumor ^s,^*	0.21	6.81	0.71	2.53 × 10^3^	2.50 × 10^2^

(^S^) Source organs with experimentally derived TIAC; in walled organs, the TIAC included the content. (^t^) Target organs available for the 25g mouse model in OLINDA/EXM 2.1 from which mean absorbed dose was obtained; in walled organs, the absorbed dose is computed for the wall. (*) Absorbed dose computed with the sphere model of OLINDA/EXM 2.1. The organ %IA/g decay corrected and the normalized time-activity curves for the considered source organs are presented in the Supplementary Materials (respectively Figures S5 and S6).

**Table 4:** Mouse dosimetry comparison between [^177^Lu]Lu-1C1m-Fc conjugated with 1 or 3 DOTA. The selected organ of interested are the TEM-1 positive tumor, the liver, the kidneys, the lungs, the spleen and the uterus.

Source Organ	Absorbed Dose (mGy/MBq)	
	1 DOTA	3 DOTA
Tumor SK-N-AS	2.53 × 10^3^ ± 2.50 × 10^2^	1.82 × 10^3^ ± 3.23 × 10^2^
Liver	1.79 × 10^3^ ± 1.30 × 10^2^	2.23 × 10^3^ ± 3.99 × 10^2^
Kidneys	1.32 × 10^3^ ± 5.00 × 10^1^	7.05 × 10^2^ ± 6.03 × 10^1^
Lungs	9.83 × 10^2^ ± 2.07 × 10^2^	5.39 × 10^2^ ± 1.30 × 10^2^
Spleen	1.18 × 10^3^ ± 1.00 × 10^2^	1.20 × 10^3^ ± 7.51 × 10^1^
Uterus	1.83 × 10^3^ ± 1.40 × 10^2^	1.50 × 10^3^ ± 5.15 × 10^2^
Tumor/Liver ratio	1.4	0.8

## Data Availability

The data presented in this study are available in article or Supplementary Materials here.

## Supplementary Materials

The following are available online at https://www.mdpi.com/1999-4923/13/1/96/s1, Figure S1: Mass spectra of 1C1m-Fc conjugated with 1; 2.5; 3; 6 and 11 DOTA; Figure S2: [177Lu]Lu-1C1m-Fc immunoreactivity (IR) test on SK-N-AS cell line. The IR was not affected by the conjugation until 8.5 DOTA. A loss of immunoreactivity was observed with the highest number of DOTA; Figure S3: Pharmacokinetic modeling of [177Lu]Lu-1C1m-Fc in Balb/c nu mice bearing TEM-1 positive tumor. (a) conjugated with 1 DOTA; (b) conjugated with 3 DOTA. Error bars = SD; Figure S4: Simultaneous fit modeling of [177Lu]Lu-1C1m-Fc in Balb/c nu mice bearing TEM-1 positive tumor obtained with the pharmacokinetic model, (a1 to a4) conjugated with 1 DOTA; (b1 to b4) 1C1m-Fc conjugated with 3 DOTA. Error bars = SD; Figure S5: Organ %IA/g decay corrected at injection time; Figure S6: Normalized time-activity curves for the considered source organs. Red lines represent bi-exponential fitting curves obtained for source organs with exclusion of the tumor, stomach, urinary bladder, uterus and the salivary glands. The coefficient of determination (R2) of the fit in respect to experimental data is also reported when applicable.

## Appendix A

### Model Description

The “Kinetics” software (www.arronax-nantes.fr) allows a full description of the model and data within a single Microsoft excel worksheet. Formulas are entered in worksheet cells in a nearly natural mathematical language. The model involves 16 compartments (F1 to F16) representing blood, all measured tissues and an additional compartment (F2) for the rest of the mouse body (carcass). Tissue weights (Wi), antibody immunoreactivity (IR) and number of DOTA per antibody (DOTA) are fixed parameters. In the simultaneous fit of all data, the different preparations are represented in different “Time interrupts”, a feature, present in WinSAAM and used in the “Kinetics” software package, that allows the fixed parameters to be changed.

Transfers are assumed linear and defined in a matrix form as Ki:1*Wi*F1 (Ki:1 an adjustable rate constant and Wi the weight of tissue i) for the transfer from blood to tissue i and K1:i*Fi for the transfer back to blood (for compartment 2, the weight is unknown and omitted). Elimination is set from the blood compartment (Kel). Total blood volume cannot be identified from data because the earlier time point is 4 h. It is set to 2.2 mL (WTB). Two additional fixed parameters are DOTA, the number of DOTA per antibody, and IR, the immunoreactivity of the preparations. Then the effect of DOTA is described by setting the transfer rate from blood to liver as: K5:1*DOTA*WLi*F1 and the effect of immunoreactivity as (K3:1 + ATIR*IR)*WTu*F1 and (K15:1 + AUIR*IR)*WUt*F1 for tumor and uterus uptake respectively (increase in uptake rate for higher immunoreactivity) and as (K6:1-ASIR*IR)*WSp*F1 and (K10:1-ABIR*IR)*WBo*F1 for spleen and bone (increase in uptake rate for lower immunoreactivity).

Since the tissue contents is given as % of injected activity per g (%IA/g) and each measured tissue is represented by the content of a compartment plus an adjustable fraction (BTu to BBl) of blood (F1). The injected activity is set to 100 and the tissue contents are normalized by the tissue weights (WTu to WBl).

The calculations (simulations and parameter adjustment) are triggered by the user from the Excel worksheet through a VBA macro that call advanced functions of a dynamic linked library written in Pascal. The results are returned to the same Excel worksheet. Reasonably close starting values for the 49 adjustable parameters and supervised fitting are necessary.

**Table A1:** Simultaneous fit modeling.

	1 DOTA			3 DOTA			Simultaneous Fit				1 DOTA			3 DOTA			Simultaneous Fit		
Parameter	Value	±	SD	Value	±	SD	Value	±	SD	Parameter	Value	±	SD	Value	±	SD	Value	±	SD
K2:1	1.01 × 10^−1^	±	7.31 × 10^−3^	2.67 × 10^−1^	±	2.92 × 10^−2^	1.08 × 10^−1^	±	1.65 × 10^−2^	BTu	3.68 × 10^−2^	±	1.98 × 10^−3^	1.41 × 10^−2^	±	1.88 × 10^−3^	4.74 × 10^−3^	±	3.21 × 10^−3^
K1:2	7.65 × 10^−2^	±	4.28 × 10^−3^	2.76 × 10^−1^	±	3.28 × 10^−2^	8.11 × 10^−2^	±	1.01 × 10^−2^	BLu	2.75 × 10^−2^	±	1.25 × 10^−3^	2.75 × 10^−2^	±	1.54 × 10^−3^	2.38 × 10^−2^	±	3.03 × 10^−3^
K3:1	1.63 × 10^−2^	±	7.06 × 10^−4^	2.37 × 10^−2^	±	1.30 × 10^−3^	9.27 × 10^−3^	±	2.87 × 10^−3^	BLi	2.49 × 10^−1^	±	8.67 × 10^−3^	2.75 × 10^−1^	±	1.46 × 10^−2^	1.83 × 10^−1^	±	2.05 × 10^−2^
K1:3	1.07 × 10^−2^	±	6.96 × 10^−4^	2.31 × 10^−2^	±	1.20 × 10^−3^	2.33 × 10^−2^	±	2.44 × 10^−3^	BSp	1.65 × 10^−2^	±	7.39 × 10^−4^	1.68 × 10^−2^	±	9.29 × 10^−4^	1.51 × 10^−2^	±	1.73 × 10^−3^
K4:1	3.85 × 10^−3^	±	4.44 × 10^−4^	3.32 × 10^−3^	±	7.04 × 10^−4^	7.97 × 10^−3^	±	1.87 × 10^−3^	BKi	4.85 × 10^−2^	±	2.12 × 10^−3^	5.38 × 10^−2^	±	2.78 × 10^−3^	4.93 × 10^−2^	±	5.31 × 10^−3^
K1:4	2.06 × 10^−2^	±	2.24 × 10^−3^	3.03 × 10^−2^	±	5.00 × 10^−3^	3.98 × 10^−2^	±	8.80 × 10^−3^	BHe	1.43 × 10^−2^	±	6.17 × 10^−4^	1.54 × 10^−2^	±	8.02 × 10^−4^	1.36 × 10^−2^	±	1.63 × 10^−3^
K5:1	3.18 × 10^−3^	±	1.02 × 10^−4^	9.90 × 10^−3^	±	6.29 × 10^−4^	7.62 × 10^−3^	±	5.80 × 10^−4^	BMu	2.07 × 10^−3^	±	1.53 × 10^−4^	2.62 × 10^−3^	±	1.96 × 10^−4^	2.23 × 10^−3^	±	3.70 × 10^−4^
K1:5	0.00	±	NA	1.25 × 10^−2^	±	9.88 × 10^−4^	1.76 × 10^−2^	±	1.94 × 10^−3^	BBo	2.35 × 10^−3^	±	1.16 × 10^−4^	2.99 × 10^−3^	±	1.53 × 10^−4^	2.49 × 10^−3^	±	2.90 × 10^−4^
K6:1	4.20 × 10^−3^	±	3.06 × 10^−4^	7.83 × 10^−3^	±	6.85 × 10^−4^	6.36 × 10^−2^	±	6.77 × 10^−3^	BSt	9.96 × 10^−3^	±	5.69 × 10^−4^	8.55 × 10^−3^	±	6.54 × 10^−4^	8.84 × 10^−3^	±	1.34 × 10^−3^
K1:6	8.50 × 10^−3^	±	9.92 × 10^−4^	1.95 × 10^−2^	±	1.70 × 10^−3^	1.77 × 10^−2^	±	2.60 × 10^−3^	BSi	6.85 × 10^−2^	±	3.37 × 10^−3^	7.03 × 10^−2^	±	4.05 × 10^−3^	6.69 × 10^−2^	±	8.29 × 10^−3^
K7:1	3.68 × 10^−3^	±	2.50 × 10^−4^	3.54 × 10^−3^	±	3.78 × 10^−4^	4.91 × 10^−3^	±	6.68 × 10^−4^	BCo	4.14 × 10^−2^	±	2.00 × 10^−3^	4.14 × 10^−2^	±	2.41 × 10^−3^	3.74 × 10^−2^	±	4.94 × 10^−3^
K1:7	4.72 × 10^−3^	±	8.76 × 10^−4^	1.50 × 10^−2^	±	1.66 × 10^−3^	1.32 × 10^−2^	±	2.45 × 10^−3^	BOv	7.59 × 10^−3^	±	2.54 × 10^−4^	2.48 × 10^−3^	±	2.32 × 10^−4^	3.33 × 10^−3^	±	5.24 × 10^−4^
K8:1	1.03 × 10^−3^	±	2.24 × 10^−4^	1.37 × 10^−3^	±	3.52 × 10^−4^	3.28 × 10^−3^	±	1.09 × 10^−3^	BUt	2.65 × 10^−2^	±	1.14 × 10^−3^	1.08 × 10^−2^	±	7.19 × 10^−4^	1.17 × 10^−2^	±	1.57 × 10^−3^
K1:8	1.58 × 10^−2^	±	3.35 × 10^−3^	2.42 × 10^−2^	±	4.96 × 10^−3^	3.68 × 10^−2^	±	1.12 × 10^−2^	BBl	7.88 × 10^−4^	±	5.44 × 10^−5^	5.88 × 10^−4^	±	6.41 × 10^−5^	6.32 × 10^−4^	±	1.15 × 10^−4^
K9:1	1.53 × 10^−3^	±	1.00 × 10^−4^	9.49 × 10^−4^	±	1.01 × 10^−4^	1.46 × 10^−3^	±	2.07 × 10^−4^	Kel	3.51 × 10^−2^	±	5.48 × 10^−4^	4.23 × 10^−2^	±	6.64 × 10^−4^	4.53 × 10^−2^	±	1.54 × 10^−3^
K1:9	1.07 × 10^−2^	±	1.08 × 10^−3^	1.53 × 10^−2^	±	1.85 × 10^−3^	1.49 × 10^−2^	±	2.92 × 10^−3^	ATIR							2.57 × 10^−2^	±	3.74 × 10^−3^
K10:1	1.51 × 10^−3^	±	1.33 × 10^−4^	1.14 × 10^−3^	±	1.32 × 10^−4^	1.09 × 10^−2^	±	1.58 × 10^−3^	ASIR							6.37 × 10^−2^	±	7.45 × 10^−3^
K1:10	4.42 × 10^−3^	±	1.09 × 10^−3^	6.08 × 10^−3^	±	1.45 × 10^−3^	7.62 × 10^−3^	±	2.48 × 10^−3^	ABIR							1.05 × 10^−2^	±	1.75 × 10^−3^
K11:1	1.26 × 10^−3^	±	1.04 × 10^−4^	9.95 × 10^−4^	±	1.02 × 10^−4^	1.78 × 10^−3^	±	2.62 × 10^−4^	AUIR							1.68 × 10^−3^	±	2.31 × 10^−3^
K1:11	9.67 × 10^−3^	±	1.26 × 10^−3^	1.46 × 10^−2^	±	1.65 × 10^−3^	1.80 × 10^−2^	±	3.28 × 10^−3^										
K12:1	9.21 × 10^−4^	±	9.60 × 10^−5^	8.57 × 10^−4^	±	1.10 × 10^−4^	1.49 × 10^−3^	±	2.57 × 10^−4^										
K1:12	6.19 × 10^−3^	±	1.36 × 10^−3^	9.86 × 10^−3^	±	1.75 × 10^−3^	1.30 × 10^−2^	±	3.08 × 10^−3^										
K13:1	7.66 × 10^−4^	±	7.69 × 10^−5^	8.51 × 10^−4^	±	1.06 × 10^−4^	1.72 × 10^−3^	±	2.76 × 10^−4^										
K1:13	2.28 × 10^−3^	±	1.21 × 10^−3^	1.02 × 10^−2^	±	1.73 × 10^−3^	1.52 × 10^−2^	±	3.24 × 10^−3^										
K14:1	1.49 × 10^−3^	±	6.42 × 10^−5^	3.81 × 10^−3^	±	2.60 × 10^−4^	4.80 × 10^−3^	±	5.90 × 10^−4^										
K1:14	0.00	±	NA	1.68 × 10^−2^	±	1.25 × 10^−3^	1.57 × 10^−2^	±	2.50 × 10^−3^										
K15:1	6.36 × 10^−3^	±	3.76 × 10^−4^	9.85 × 10^−3^	±	5.63 × 10^−4^	9.90 × 10^−3^	±	1.87 × 10^−3^										
K1:15	2.98 × 10^−3^	±	7.11 × 10^−4^	1.27 × 10^−2^	±	9.37 × 10^−4^	1.01 × 10^−2^	±	1.51 × 10^−3^										
K16:1	5.86 × 10^−3^	±	2.74 × 10^−4^	6.82 × 10^−3^	±	4.16 × 10^−4^	6.38 × 10^−3^	±	5.53 × 10^−4^										
K1:16	8.19 × 10^−3^	±	7.14 × 10^−4^	1.75 × 10^−2^	±	1.20 × 10^−3^	1.10 × 10^−2^	±	1.64 × 10^−3^										
