# Supplementary material for: Impact of DOTA Conjugation on Pharmacokinetics and Immunoreactivity of [177Lu]Lu-1C1m-Fc, an Anti TEM-1 Fusion Protein Antibody in a TEM-1 Positive Tumor Mouse Model
Source: Pharmaceutics. 2021 Jan 13;13(1):96. doi: 10.3390/pharmaceutics13010096 (PMC7828678; doi:10.3390/pharmaceutics13010096)
Supplement: Supplementary file 1 [file pharmaceutics-13-00096-s001.pdf]

pharmaceutics

MDPI

Supplementary Materials: Impact of DOTA Conjugation on Pharmacokinetics and Immunoreactivity of [177Lu]Lu-1C1m-Fc, an anti TEM-1 Antibody Fusion Protein in a TEM-1 Positive Tumor Mice Model

Judith Anna Delage, Alain Faivre-Chauvet, Jacques Barbet, Julie Katrin Fierle, Niklaus Schaefer, Georges Coukos, David. Viertl, Steven Mark Dunn, Silvano Gnesin, John O. Prior

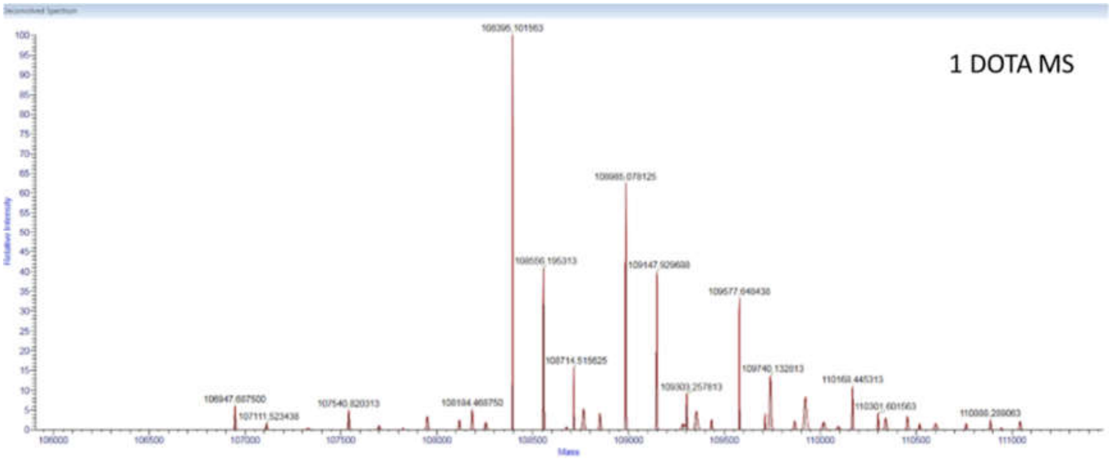

DARavr = (0 × 100+1 × 60+2 × 40+3 × 15+4 × 5)/(100+60+40+15+5) = 0.93 ~ **1**

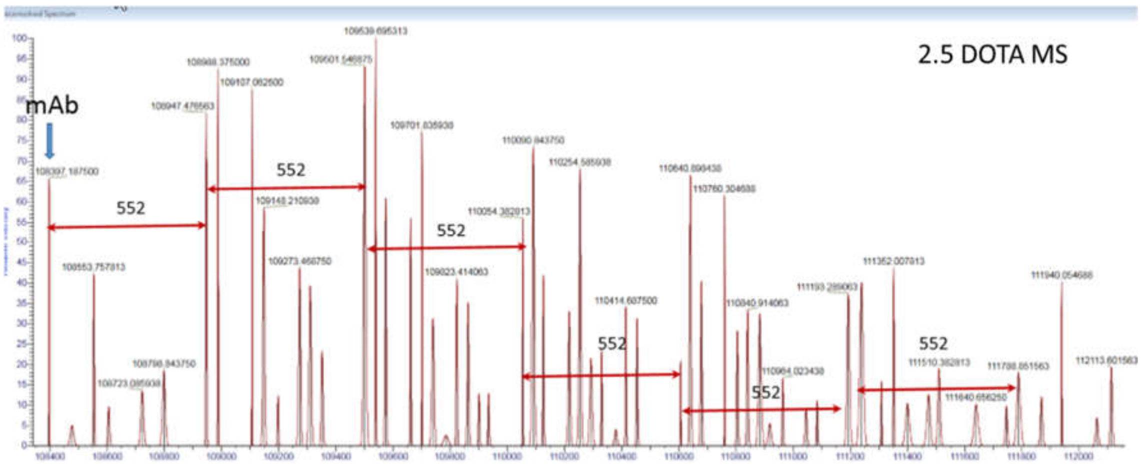

DARavr = (0 × 20+1 × 50+2 × 100+3 × 90+4 × 55+5 × 50+6 × 20+7 × 10)/(20+50+100+90+55+50+20+10)= 2.43 ~ **2.5**

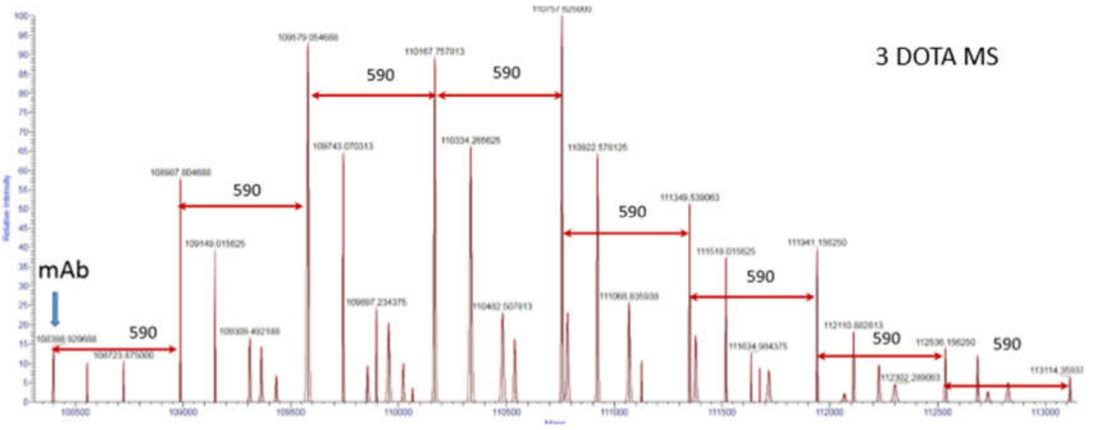

DARavr = (0 × 15+1 × 60+2 × 95+3 × 90+4 × 95+5 × 55+6 × 40+7 × 15+8 × 5)/(15+60+95+90+95+55+40+15+5) = 3.2 ~ **3**

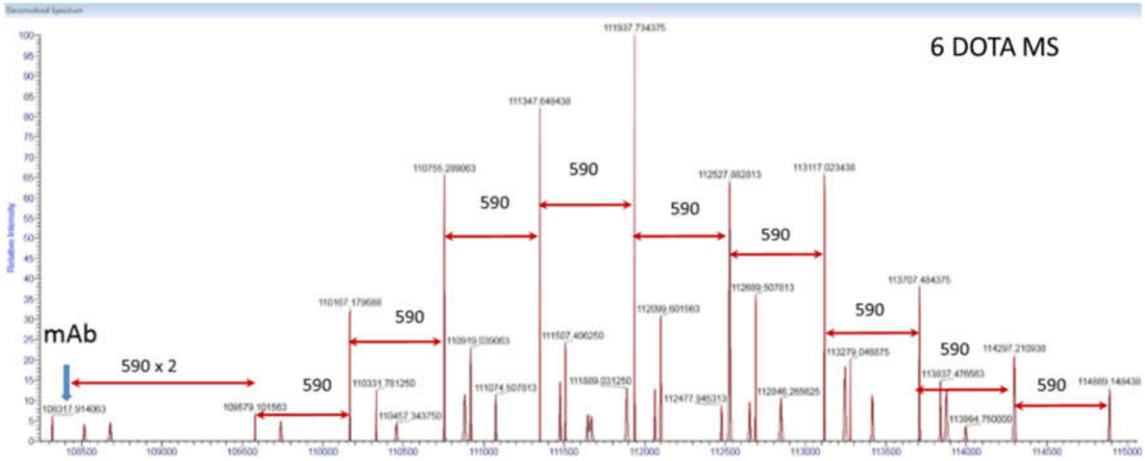

DARavr = (0 × 10+2 × 10+3 × 35+4 × 65+5 × 80+6 × 100+7 × 65+8 × 65+9 × 35+10 × 25+11 × 15)/(10+10+35+65+80+100+65+65+35+25+15) = 6.1

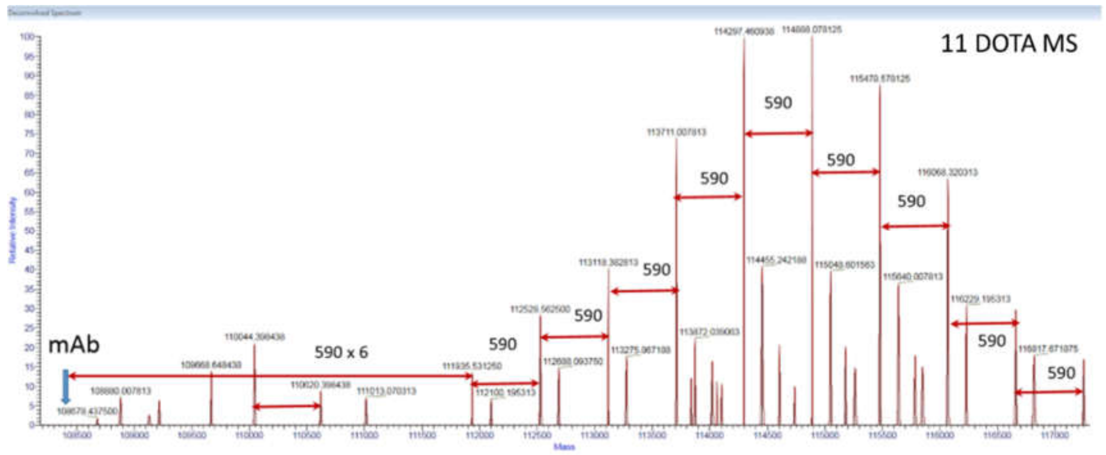

DAR avr = (6 × 15+7 × 25+8 × 40+9 × 75+10 × 100+11 × 100+12 × 85+13 × 60+14 × 30+15 × 15)/(15+25+40+75+100+100+85+60+30+15) = 10.6 ~ **11**

Figure S1. Mass spectra of 1C1m-Fc conjugated with 1; 2.5; 3; 6 and 11 DOTA.

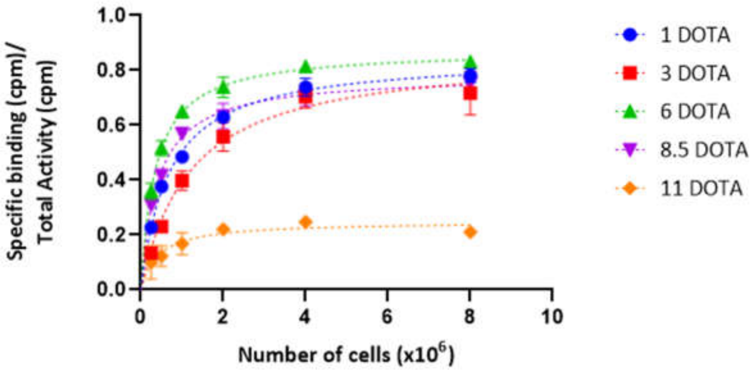

Figure 2. [^{177}Lu]Lu-1C1m-Fc immunoreactivity (IR) test on SK-N-AS cell line. The IR was not affected by the conjugation until 8.5 DOTA. A loss of immunoreactivity was observed with the highest number of DOTA.

a

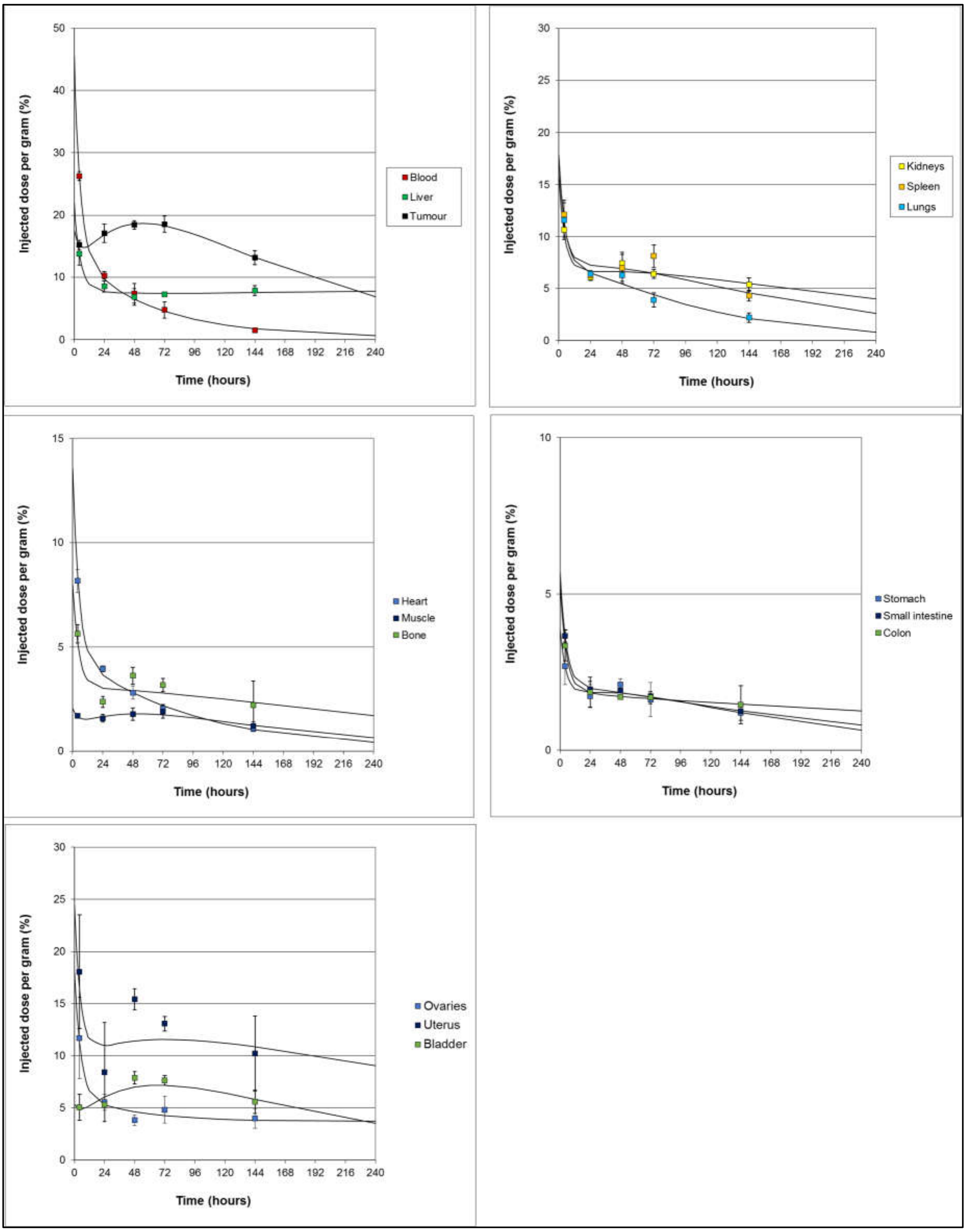
Injected dose per gram (%)
Time (hours)
0 24 48 72 96 120 144 168 192 216 240
Blood
Liver
Tumour
Kidneys
Spleen
Lungs
Heart
Muscle
Bone
Stomach
Small intestine
Colon
Ovaries
Uterus
Bladder

b

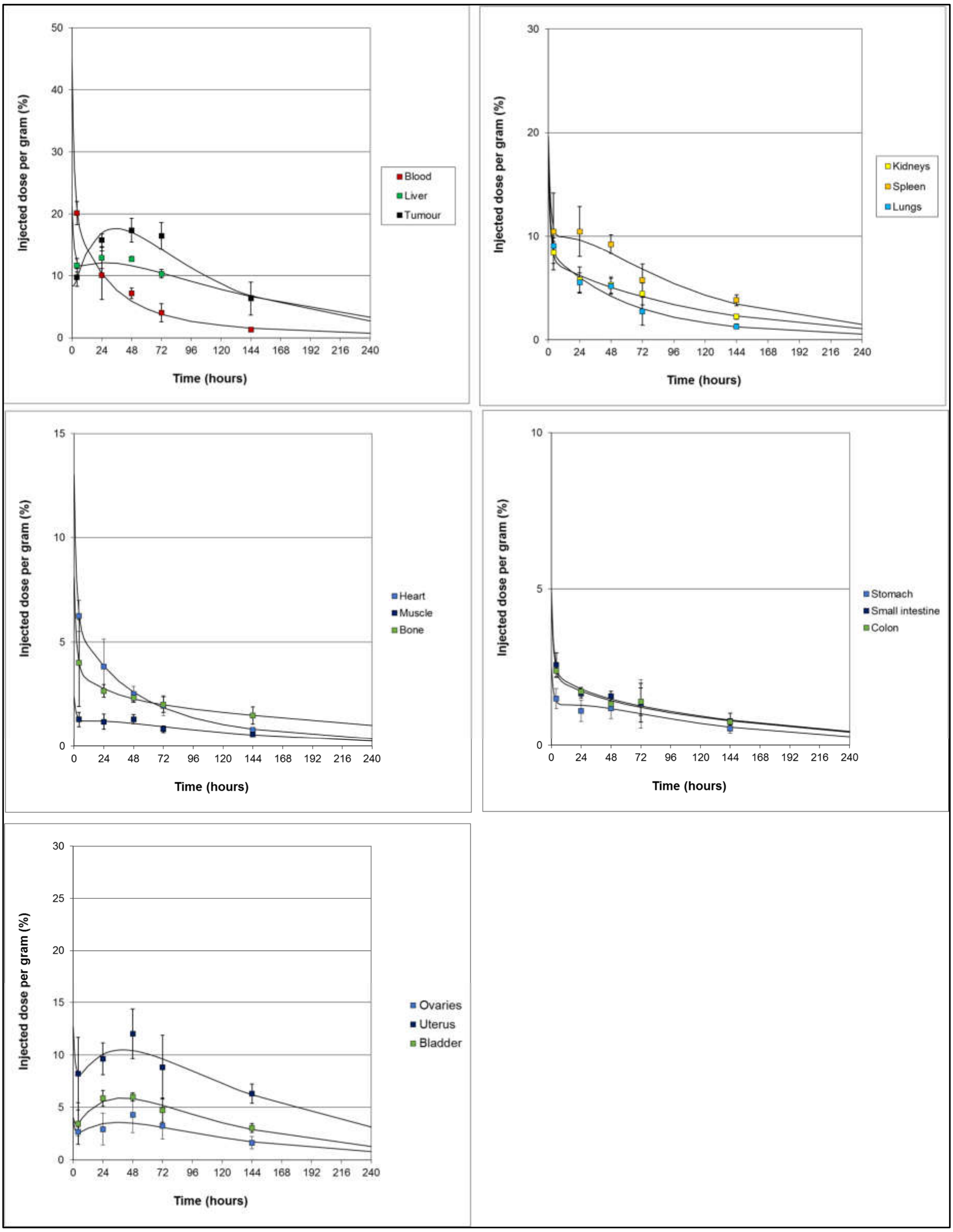

Figure S3. Pharmacokinetic modeling of [177Lu]Lu-1C1m-Fc in Balb/c nu mice bearing TEM-1 positive tumor. **(a)** conjugated with 1 DOTA; **(b)** conjugated with 3 DOTA. Error bars = SD.

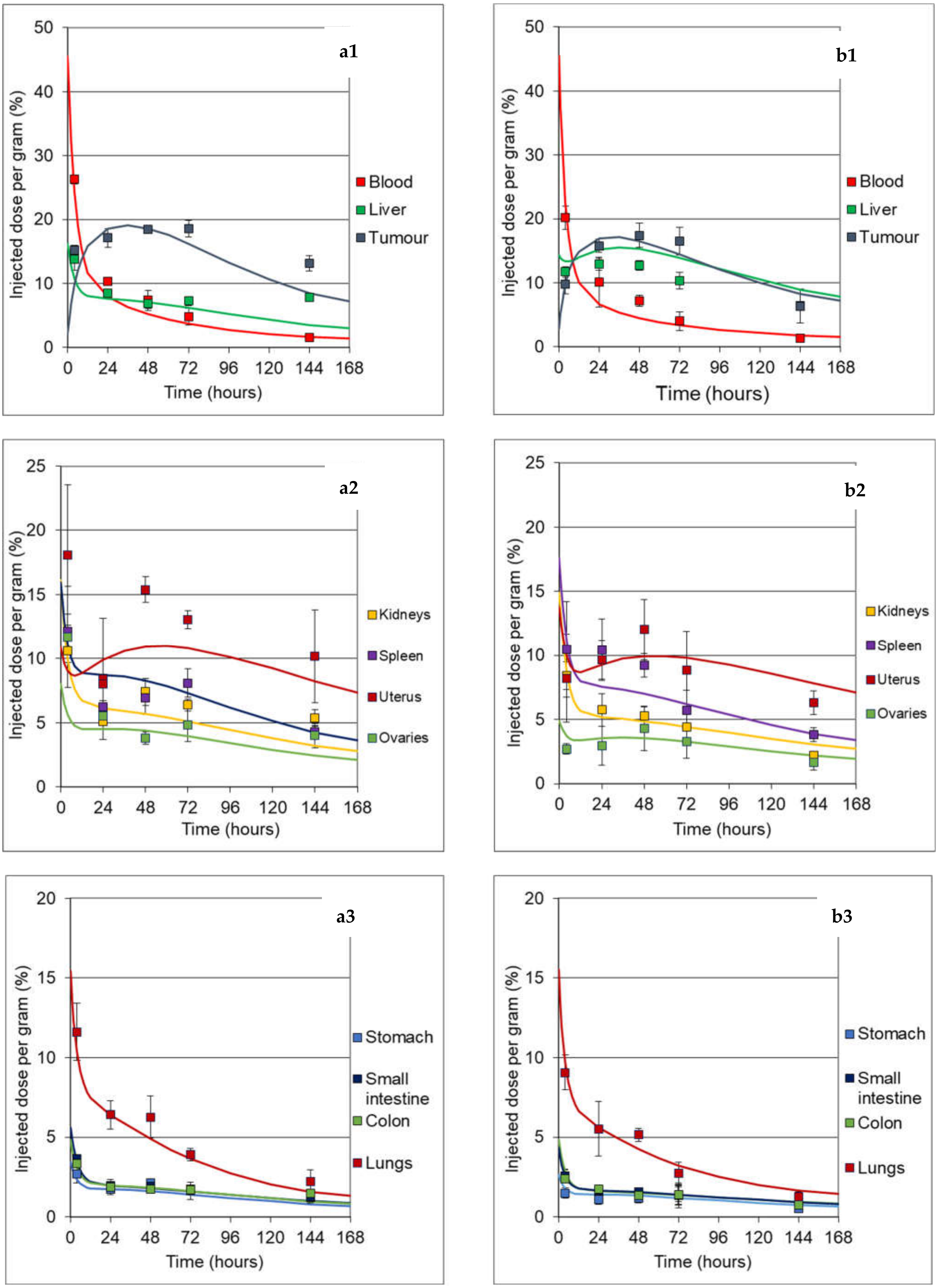
a1
Injected dose per gram (%)
Time (hours)
Blood
Liver
Tumour
b1
Injected dose per gram (%)
Time (hours)
Blood
Liver
Tumour
a2
Injected dose per gram (%)
Time (hours)
Kidneys
Spleen
Uterus
Ovaries
b2
Injected dose per gram (%)
Time (hours)
Kidneys
Spleen
Uterus
Ovaries
a3
Injected dose per gram (%)
Time (hours)
Stomach
Small intestine
Colon
Lungs
b3
Injected dose per gram (%)
Time (hours)
Stomach
Small intestine
Colon
Lungs

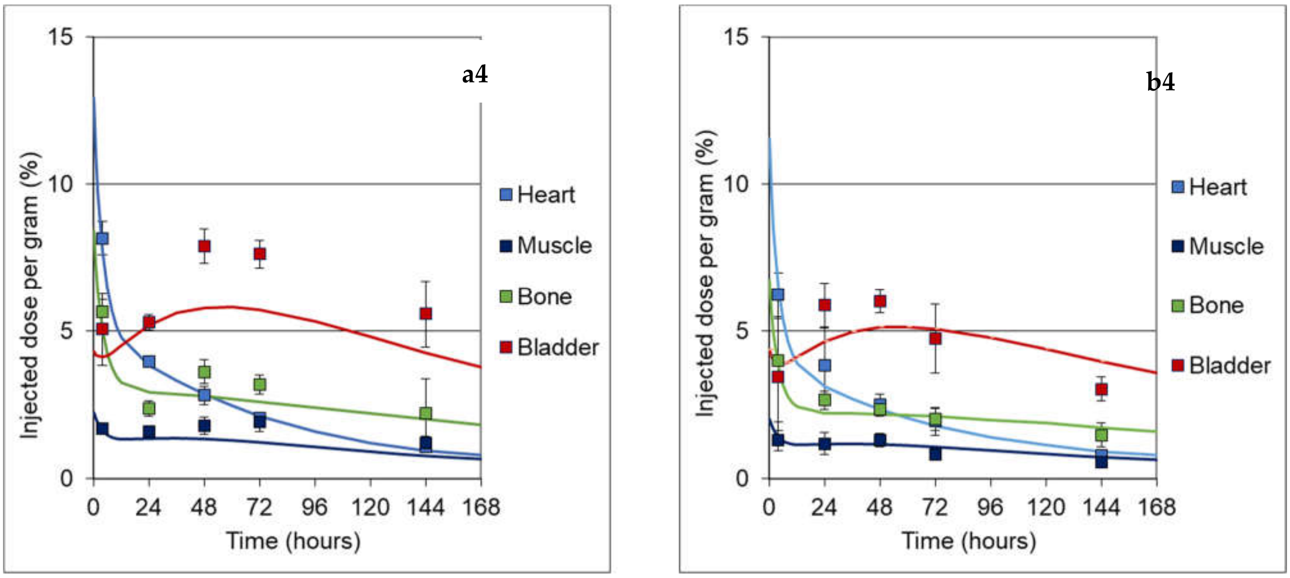

Figure S4. Simultaneous fit modeling of [^{177}Lu]Lu-1C1m-Fc in Balb/c nu mice bearing TEM-1 positive tumor obtained with Table 1. to a4) conjugated with 1 DOTA; (b1 to b4) 1C1m-Fc conjugated with 3 DOTA. Error bars = SD.

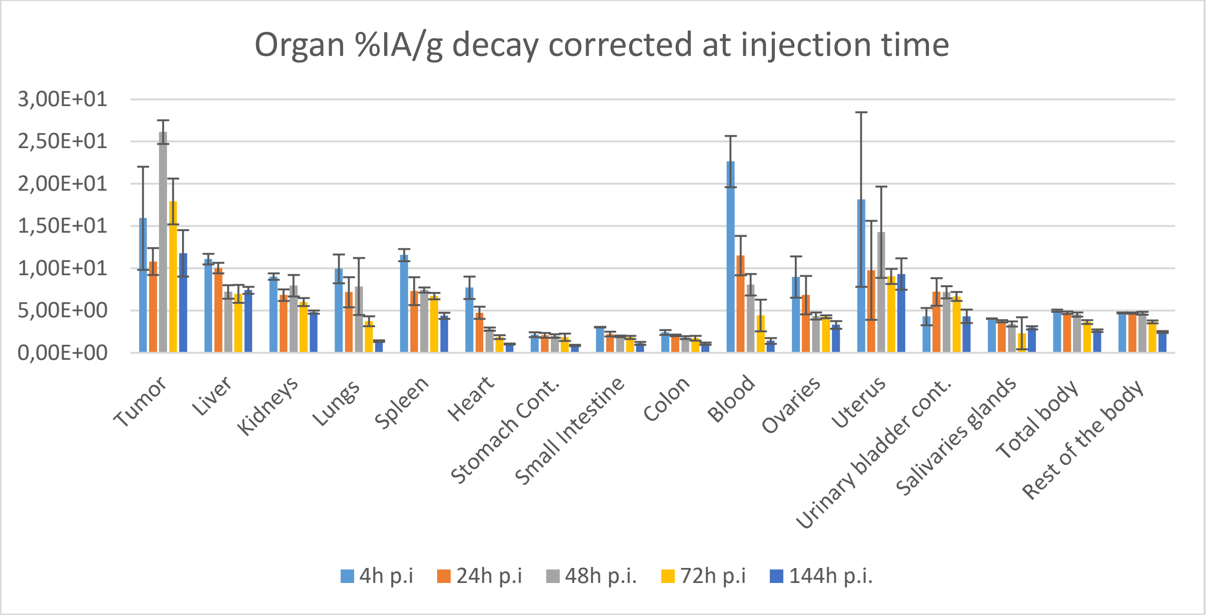

Figure S5. Organ %IA/g decay corrected at injection time.

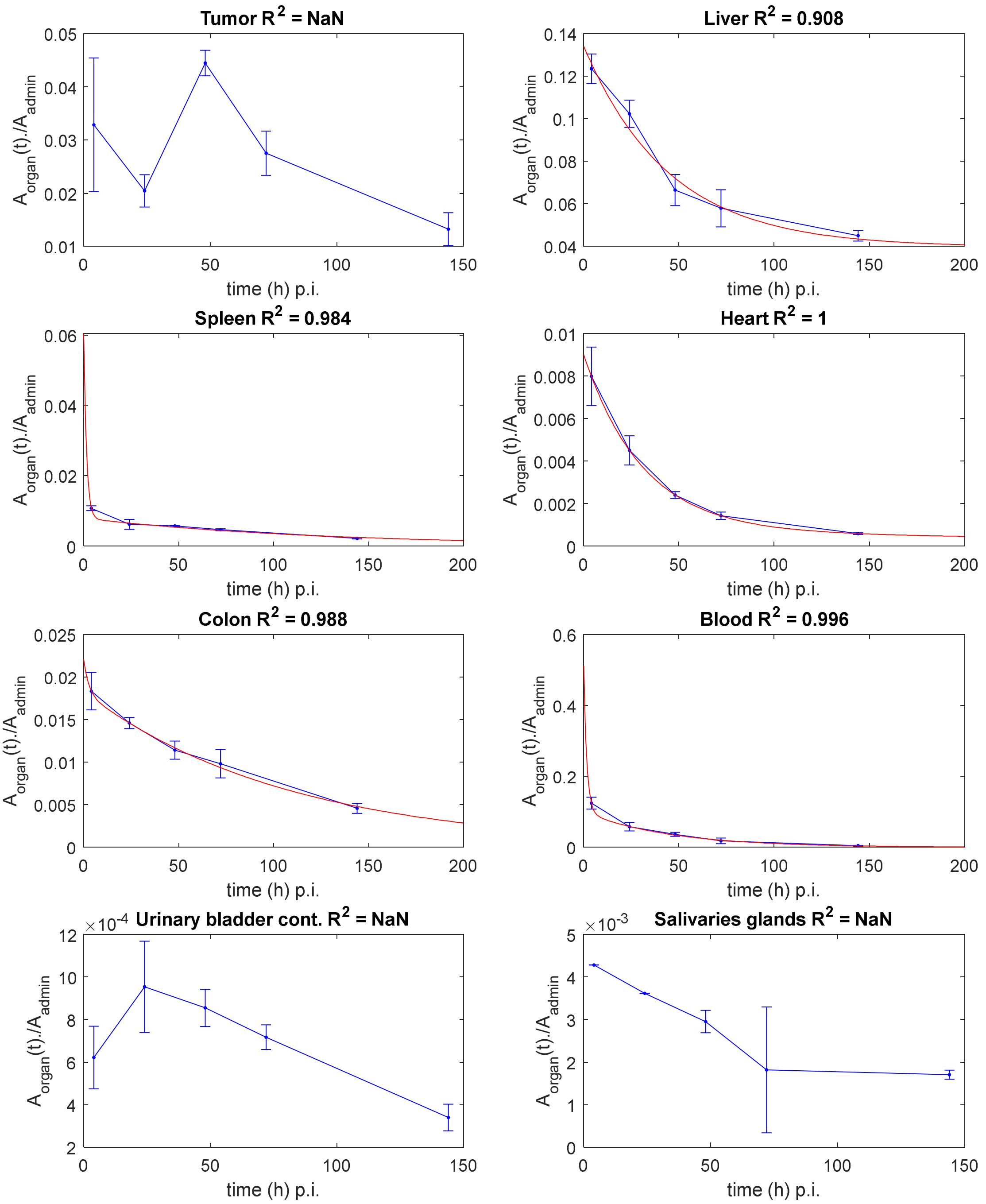
Tumor R2 = NaN
Liver R2 = 0.908
Spleen R2 = 0.984
Heart R2 = 1
Colon R2 = 0.988
Blood R2 = 0.996
×10-4 Urinary bladder cont. R2 = NaN
×10-3 Salivaries glands R2 = NaN
Aorgan(t)./Aadmin
time (h) p.i.

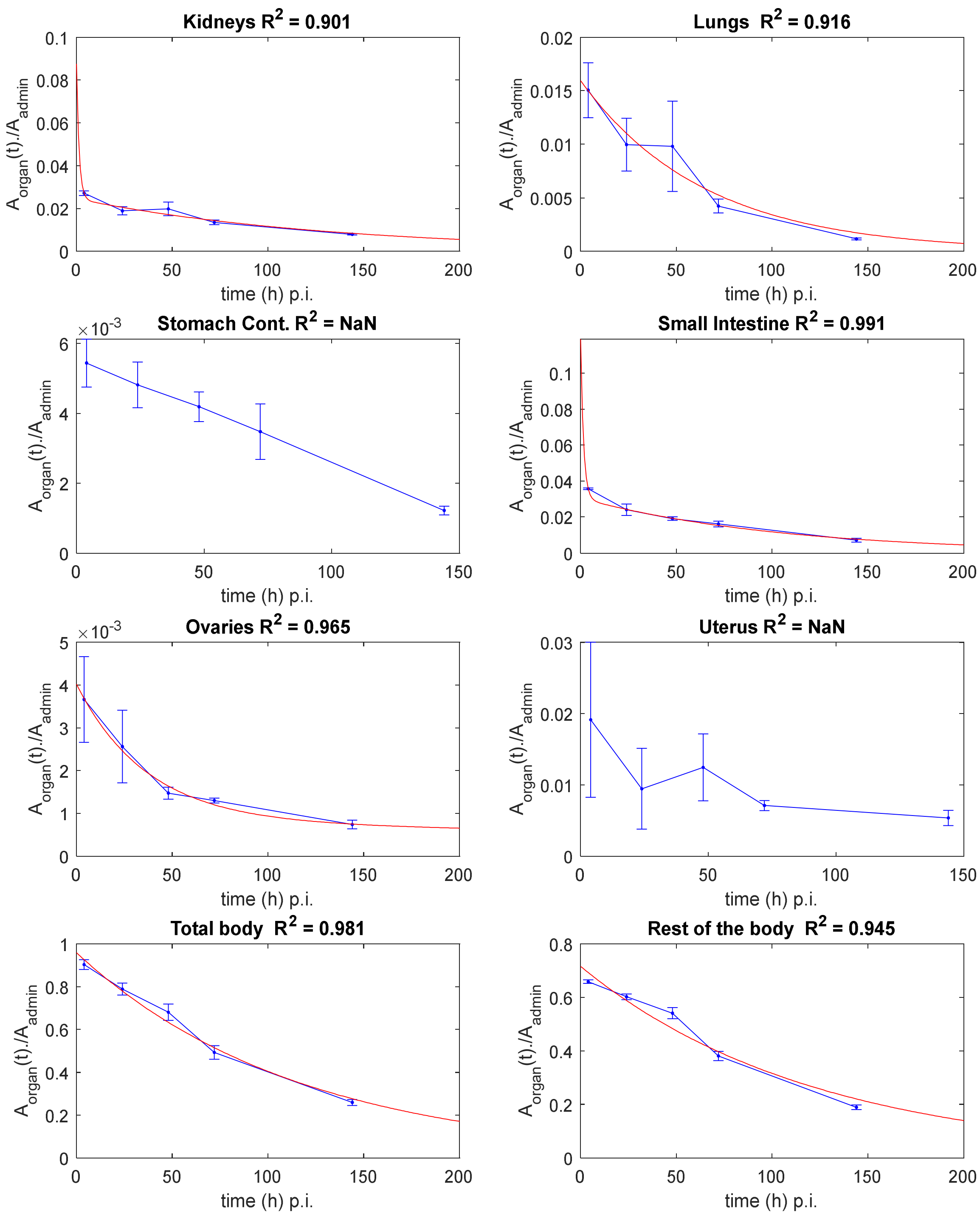

Figure S6. Normalized time-activity curves for the considered source organs. Red lines represent bi-exponential fitting curves obtained for source organs with exclusion of the tumor, stomach, urinary bladder, uterus and the salivary glands. The coefficient of determination (R^2) of the fit in respect to experimental data is also reported when applicable.
